# Antibacterial activity of clove (*Syzygium aromaticum*) and cinnamon (*Cinnamomum burmannii*) essential oil against extended-spectrum β-lactamase-producing bacteria

**DOI:** 10.14202/vetworld.2021.2206-2211

**Published:** 2021-08-25

**Authors:** Elgio Venanda Ginting, Endah Retnaningrum, Dyah Ayu Widiasih

**Affiliations:** 1 Faculty of Veterinary Medicine, Universitas Gadjah Mada, Yogyakarta, Indonesia; 2 Faculty of Biology, Universitas Gadjah Mada, Yogyakarta, Indonesia

**Keywords:** cinnamon (*Cinnamomum burmannii*), clove (*Syzygium aromaticum*), extended-spectrum β-lactamase, essential oils, *Escherichia coli*, *Klebsiella pneumoniae*

## Abstract

**Background and Aim:**

Extended-spectrum β-lactamase (ESBL) is an enzyme produced by the family of *Enterobacteriaceae*, especially *Escherichia coli* and *Klebsiella pneumoniae*, which can hydrolyzeβ-lactam antibiotics, such as penicillins, cephalosporins, cephamycin, and carbapenem. ESBL-producing bacteria are widely distributed from farms to slaughterhouses until food products originating from animals are available in the market, which plays an important role as a pathway for the exposure and transmission of ESBL-producing bacteria from food products of animal origin to humans. This study aimed to determine the antibacterial activity of *Syzygium aromaticum* (clove) and *Cinnamomum verum* (cinnamon) essential oils against strains resistant to ESBL-producing *E. coli* and *K. pneumoniae* isolates.

**Materials and Methods:**

The antibacterial activity of clove and cinnamon essential oils was tested against three strains of tested bacteria using the disk diffusion method. The minimum inhibitory concentration (MIC) of clove and cinnamon essential oils was determined using the broth microdilution method. The minimumbactericidal concentration (MBC) was determined using the MIC. Morphological changes on each tested bacteria were observed through scanning electron microscopy (SEM).

**Results:**

Both essential oils exhibited inhibitory effects toward all test organisms, indicated by inhibition zones around the disk. The MIC values of clove essential oil were 0.078% (v/v) for all tested bacteria, whereas the MICs of cinnamon essential oil ranged from 0.039% (v/v) to 0.156% (v/v) for all tested bacteria. MBC values of clove and cinnamon essential oils ranged from 0.078% (v/v) to 0.156% (v/v) for all tested bacteria. There were morphological changes in each tested bacterial cell that was observed through SEM. Each tested bacteria treated with clove and cinnamon essential oils showed shrinkage and cells lysis.

**Conclusion:**

It was concluded that clove and cinnamon essential oils have emerged as effective antibacterial agents by showing high antibacterial activity against ESBL-producing *E. coli* and *K. pneumoniae* isolates, as evidenced by the inhibition zone diameter and MIC value.

## Introduction

Extended-spectrum β-lactamase (ESBL)-producing bacteria developed as a zoonotic issue, resistant to various antibiotics. It is currently the main challenge in treating diseases, which bacterial agents cause. Antibacterial resistance and food safety issues have become a global concern recently due to their major impact on public health and the global economy.

Resistant bacteria that contaminate food raise concerns about food safety. Therefore, research to find new antibacterial agents from natural sources is necessary [1]. Natural antibacterials derived from plant and bacterial metabolites as food additives are currently being promoted to replace synthetic antibacterials [2-4]. This can be used to protect the safety of consumers and food products. In the food industry, antibacterials are used for two essential reasons: Ensuring the preservation of food products and inhibiting the growth of microorganisms in food products [5].

The largest antibacterial components contained in *Syzygium aromaticum* (clove) and *Cinnamomum verum* (cinnamon) essential oils are eugenol in clove and cinnamaldehyde in cinnamon, which have had antibacterial activity against foodborne pathogens [6-8]. *Klebsiella pneumoniae* cells treated with eugenol have shown cell membrane destruction and cell swelling [9]. Cinnamaldehyde has been reported to inhibit *Escherichia coli*, increase cell membrane permeability, and oxidize the cell membrane [10]. Eugenol in clove and cinnamaldehyde in cinnamon can cause lysis to bacterial membranes and inhibit β-lactamase production in *E. coli*, as previously reported [11]. Previous studies also reported that clove and cinnamon essential oils can enhance the shelf life of chicken meat [12,13]. In this research, we test the antibacterial activity of clove and cinnamon essential oils against ESBL-producing bacteria.

This study aimed to determine the antibacterial activity of clove and cinnamon essential oils against strains resistant to ESBL-producing *E. coli* and *K. pneumoniae* isolates.

## Materials and Methods

### Ethical approval

This study did not involve living creatures, either both animals and humans as research subjects, so it does not require ethical approval.

### Study period and location

This study was conducted from July to December 2020. The antibacterial activity of clove and cinnamon essential oils were tested at the Veterinary Public Health Laboratory, Faculty of Veterinary Medicine, and Microbiology Laboratory Faculty of Biology, Universitas Gadjah Mada, Indonesia.

### Test organisms

Two isolates standard of *E. coli* (ATCC 25922) and *K. pneumoniae* (ATCC 700603) obtained from the culture collection at Microbiology Laboratory, Faculty of Medicine, Gadjah Mada University, were used in this study. In addition, *E. coli-*producing ESBL isolates originating from chicken meat in traditional markets (CM ESBL-EC) was obtained from the culture collection at the Veterinary Public Health Laboratory, Faculty of Veterinary Medicine, Gadjah Mada University. All cultures were stored in the slant agar at 4°C, and it was used as a stock culture for 14 days.

### Identification and authentication of the plants used in this research

The clove and cinnamon used in this research have been identified at the Laboratory of Plant Systematic, Faculty of Biology, Universitas Gadjah Mada, Yogyakarta, Indonesia, by the botanist Ludmilla Fitri Untari, S.Si., M.Sc.

### Isolation and preparation of clove and cinnamon essential oils

Clove bud was obtained from farmers in Girimulyo Village, Kulon Progo Regency, Yogyakarta, whereas the cinnamon bark was obtained from Kledung Village, Temanggung Regency, Central Java. Clove and cinnamon essential oils were distilled using the steam distillation method, according to Masango [14]. First, 1 kg of clove flowers and 2 kg of cinnamon bark were crushed. Steam distillation was done by placing the crushed clove flowers and cinnamon bark on the shelf, and then, water was boiled under the kettle shelf. The kettle was heated using an electric stove. The distillation time took 4 h, and it was measured from the first condensate drop. The material and water were separated using a sieve (filter). A sieve was placed under the water surface to avoid direct contact between the water and material, leaving only the essential oils.

### Chemical composition of the essential oil obtained

For the chemical composition of clove buds and cinnamon bark essential oils, we refer to previous research using the same plant from the exact origin [15,16], as shown in Tables-1 and 2.

**Table-1:** Chemical composition (relative percentage, extreme values) of clove bud (*Syzygium aromaticum*).

Compounds	Percentage
Methyl salicylate	0.04-0.16
Chavicol	0.13-0.18
Eugenol	77.32-82.36
a-Copaene	0.17-0.27
methyl eugenol	0.04-0.08
β-Caryophyllene	5.34-8.64
Isoeugenol	0.02-0.24
α-Humulene	0.65-1.04
Eugenyl acetate	8.61-10.55
Caryophyllene oxide	0.06-0.32

**Table-2:** Chemical composition of cinnamon bark (*Syzygium aromaticum*).

Compounds	Percentage
Styrene	0.62
IR-a-pinene	1.68
Camphene	0.88
Benzaldehyde	1.21
β-Pinene	0.64
Eucalyptol	3.46
Benzene propanol	2.11
Borneol	0.70
3-Cyclohexen-1ol	1.04
p-Ment-1-eu-8-ol	1.04
Cinnamaldehyde	61.16
Benzene propanol	0.89
Copaene	3.54
Caryophyllene	0.75
2H-1-Benzopyran-2-one	1.16
2-Propen-1-ol, 3-phenyl-acetat	1.46
a-Caryophyllene	0.55
Naphthalene	8.94
a-Cubebene	0.00
Caryophyllene oxide	0.98

### Antibacterial sensitivity testing

The antibacterial activity of clove and cinnamon essential oils was tested on the basis of the method used by Balouiri *et al*. [17] and Gulfraz *et al*. [18] with modification. First, each bacterium was subcultured in nutrient broth (Merck, Darmstadt, Germany) for 24 h at 37°C. Then, 150 μL of standardized inoculum (10^8^ colony-forming unit [CFU]/mL; 0.5 McFarland) of each bacterium tested was spread onto sterile Mueller-Hinton agar (MHA) (Merck, Darmstadt, Germany) to achieve confluent growth.

The Whatman blank disks with a diameter of 6 mm were sterilized; then, 10 L essential oil was injected, which was diluted with dimethyl sulfoxide (DMSO) to achieve reduction concentration ranged between 40%, 20%, and 10% (v/v) and placed on the agar surface under aseptic conditions. Sterile DMSO served as the negative control, and chloramphenicol disks (Oxoid, Dublin, Ireland) were a positive control. The inoculated plate was incubated at 37°C during 18-24 h. Then, antibacterial activity was evaluated by measuring the zone of inhibition (mm) against the test organism. Each antibacterial activity test was repeated thrice.

### Determination of minimum inhibitory concentration (MIC) and minimum bactericidal concentration (MBC) of cloves and cinnamon essential oils

To obtain the minimum concentration of clove and cinnamon essential oils to inhibit all tested bacteria (MIC), a microdilution test was conducted. The test was conducted according to Adukwu *et al*. [19]. Each tested bacteria (10^8^ CFU/mL; 0.5 McFarland) was inoculated into nutrient broth (Merck) at 50°C. Nutrient broth and cultures without essential oils added to wells of a sterile 96-well microplate were a positive control, whereas nutrient broth without cultures was the negative control.

Each essential oil was diluted with DMSO to obtain 2.5% essential oil concentrations. Then, 100 µL of each dilution of essential oils was transferred into nutrient broth containing each tested bacteria (1:1) and added to wells to give final essential oil concentrations of 1.25%, 0.625%, 0.312%, 0.156%, 0.078%, 0.039%, and 0.019% (v/v). Optical density (OD) was measured at 595 nm using a microplate reader (Bio-Rad 680XR, Hertfordshire, UK) and again after incubation for 24 h at 37°C. The MIC was determined as the lowest essential oil concentration. The OD at 24 h of the inoculum remained the same or reduced compared with that at the initial reading.

The MBC was measured by injecting 20 μL of solution from 96-well microplate which was determined as the MIC value into MHA media in Petri dish, then was spread using drigalski spatula. On the basis of the MBC/MIC ratio the antibacterial impact was considered bactericidal or bacteriostatic. The MBC values were identified as the lowest sample concentration, resulting in the initial inoculum being killed by approximately 99.9%.

### Scanning electron microscopy (SEM)

SEM was conducted following the method by Ozogul *et al*. [20], aimed at examining the further effects of clove and cinnamon essential oils on morphological changes in bacterial cell walls. The essential oils of cloves and cinnamon were put into the broth culture at the MIC of each tested bacteria and incubated for 24 h at 37°C. After incubation, each tube was centrifuged for 10 min at 4000×*g* at 4°C. The samples were then washed twice with distilled water and resuspended in 1 mL water. After that, 10 μL of the suspension was coated on the slide (1×1 cm). This sample is then placed in a vertical laminar flow biological cabinet (Telstar Class II Cabinet, Terrassa, Spain) at 25°C to be dehydrated using 10% ethanol. The prepared sample was put into a desiccator until it was coated with gold in a sputter ion coating (Quorum Technologies Ltd., East Sussex, UK) for 10 min and then observed using SEM (Quanta 650 Field Emission SEM, FEI, Hillsboro, OR, USA).

## Results

### Antibacterial activity of clove and cinnamon essential oils

The antibacterial activity of clove and cinnamon essential oils was tested using two standard strains, and ESBL-producing *E. coli* isolate originating from chicken meat showed zones of inhibition around the disk, and the diameter of zones is presented in Table-3. It was found that clove essential oil (concentration of 40%, 20%, and 10%) was potentially active against *E. coli* ATCC 25922, *K. pneumoniae* ATCC 700603, and ESBL-producing *E. coli* from chicken meat with inhibition zones ranging from 15.0±1.00 to 24.3±0.57 mm. In addition, cinnamon essential oil (concentration of 40%, 20%, and 10%) was also potentially active against *E. coli* ATCC 25922, *K. pneumoniae* ATCC 700603, and ESBL-producing *E. coli* from chicken meat with zone size ranging from 15.6±2.08 to 25.3±0.57 mm (Table-3).

**Table-3:** Inhibition zone of clove and cinnamon essential oil against each tested bacteria.

Treatment	Zone of inhibition (mm)		

	*Escherichia coli* ATCC 25922	*Klebsiella pneumoniae* ATCC 700603	*Escherichia coli* ESBL from chicken meat
Clove 10%	17.0±1.73	15.0±1.00	15.3±2.30
Clove 20%	19.3±0.57	17.3±0.57	18.0±2.00
Clove 40%	24.3±0.57	19.0±1.00	22.6±2.08
Cinnamon 10%	15.6±2.08	16.3±1.52	17.3±1.15
Cinnamon 20%	18.6±1.15	19.3±0.57	20.6±1.15
Cinnamon 40%	21.3±2.30	23.0±1.00	25.3±0.57
Control (+)*	34.6±1.15	34.0±2.00	34.0±2.00
Control (−)**	6.0±0.00	6.0±0.00	6.0±0.00

* =Positive control using *Chloramphenicol*,

** =Negative control using DMSO

### MIC and MBC of clove and cinnamon essential oils

Determination of MIC was performed to determine the antibacterial activity of clove and cinnamonessential oils. Cinnamon essential oil showed a higher antibacterial effect on ESBL-producing *E. coli* from chicken meat (lowest MIC) than clove essential oil. The MIC value of clove essential oil was 0.078% against all strains tested. Meanwhile, the MIC value of cinnamon essential oil ranged from 0.039% to 0.156%, as shown in Table-4.

**Table-4:** The MIC and MBC values of clove and cinnamon essential oils against each tested bacteria.

Bacteria	Clove			Cinnamon		
	
	MIC	MBC	MBC/MIC	MIC	MBC	MBC/MIC
*Escherichia coli ATCC 25922*	0.078%	0.078%	1	0.156%	0.156%	1
Klebsiella pneumoniae ATCC 700603	0.078%	0.156%	2	0.078%	0.156%	2
Escherichia coli ESBL from chicken meat	0.078%	0.156%	2	0.039%	0.078%	2

In this study, clove essential oil exhibited antibacterial activity. The MIC for clove essential oil was at 0.078% (v/v) for all strains tested. The MBC values of clove essential oil were at 0.078-0.156%. The lowest MBC value was observed in *E. coli* ATCC 25922, and the highest MBC value was observed in *K. pneumoniae* ATCC 700603 and ESBL-producing *E. coli* from chicken meat.

Cinnamon essential oil had MIC in the range of 0.039-0.156%, whereas its MBC was at 0.078v0.156%. The highest MIC value was observed in *E. coli* ATCC 25922, whereas the lowest MIC value was shown in ESBL-producing *E. coli* from chicken meat. The lower MBC value found for cinnamon essential oil was shown in ESBL-producing *E. coli* from chicken meat, and a higher MBC value was shown in *E. coli* ATCC 25922 and *K. pneumoniae* ATCC 700603.

From the obtained MBC/MIC ratio (Table-4), it can be noticed that clove and cinnamon essential oils indicated a bactericidal effect against all strains of bacteria tested. MBCs were equal to or 2-fold greater than the MICs. The MBC/MIC ratios ranged from 1 to 4, which were considered bactericidal [21].

### SEM

**Figure-1:**
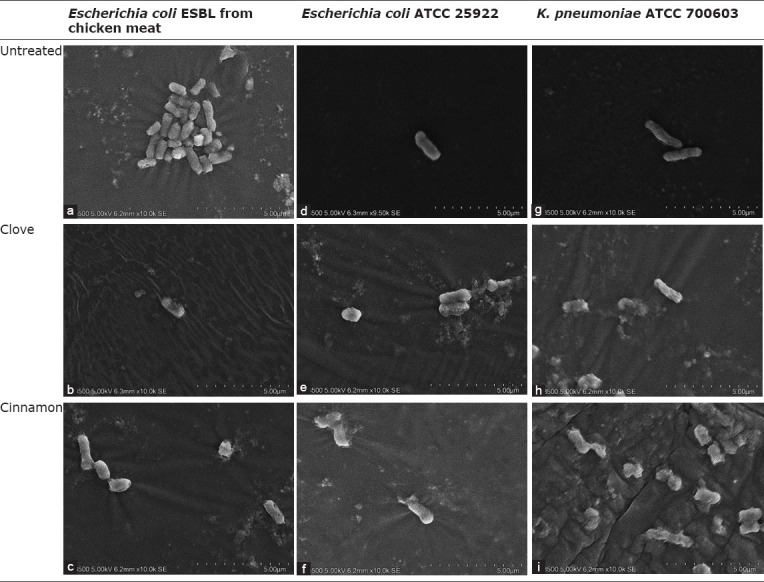
Morphological changes on each tested bacteria cells were observed by scanning electron microscopy (SEM). The untreated each tested bacteria showed normal cell shaped, while each tested bacteria treated with clove and cinnamon oil showed shrinkage and lysis cell. a: *Escherichia coli* ESBL from chicken meat untreated, b: *E. coli* ESBL from chicken meat treated with clove, c: *E. coli* ESBL from chicken meat treated with cinnamon, d: *E. coli* ATCC 25922 untreated, e: *E. coli* ATCC 25922 treated with clove, f: *E. coli* ATCC 25922 treated with cinnamon, g: *Klebsiella pneumoniae* ATCC 700603 untreated, h: *K. pneumoniae* ATCC 700603 treated with clove, i: *K. pneumoniae* ATCC 700603 treated with cinnamon.

Morphological changes in each tested bacterial cell were observed through SEM. The untreated tested bacteria showed normal cell shape, whereas each tested bacteria treated with clove and cinnamon essential oils showed shrinkage and cell lysis (Figure-1).

## Discussion

The increasing incidence of ESBL-producing bacteria is widely spread from farms to slaughterhouses to animal-based food products. It is a health concern affecting animal and human health. The strategy for managing the spread of resistant bacteria has been extensively studied, including natural compounds being one of the alternative therapies for resistant bacteria.

Clove and cinnamon essential oils were active in inhibiting *E. coli* ATCC 25922, *K. pneumoniae* ATCC 700603, and ESBL-producing *E. coli* from chicken meat at 40%, 20%, and 10% concentrations using the disk diffusion method. Essential oils with an inhibition zone diameter >7 mm against ESBL-producing bacteriaare known to have effective antibacterial activity [22]. The antibacterial activity of cloves and cinnamon has been widely reported [6,23]; in this study, we tested the antibacterial activity of clove and cinnamon essential oils against ESBL-producing bacteria as a comparison to the previous studies [8,9,23].

Clove and cinnamon essential oils have the same antibacterial effect on all bacteria tested. The MBC/MIC ratios of clove and cinnamon essential oils ranged from 1 to 2. MBC that is close to MIC demonstrated good bactericidal activity against the tested strains. The MICs of clove and cinnamon essential oils in this study were similar to those in previously reported studies [24-26], with little differences, due to several reasons such as differences in the growing environments of clove and cinnamon, different extraction methods of essential oils and maybe the use of ESBL-producing bacteria as strains tested in this study.

The effect of clove and cinnamon essential oils on *E. coli* ATCC 25922, *K. pneumoniae* ATCC 700603, and ESBL-producing *E. coli* from chicken meat observed through SEM showed changes in bacterial cell structure (Figure-1). The untreated *E. coli* ESBL cells from chicken meat, *E. coli* ATCC 25922, and *K. pneumoniae* ATCC 700603 showed intact and smooth cell membrane surfaces, whereas *E. coli* ATCC 25922 and *E. coli* ESBL cells from chicken meat treated with clove and cinnamon essential oils showed rough and damaged cell membranes; some cells were lysed and split because of wrinkle formation. Meanwhile, *K. pneumoniae* ATCC 700603 cells treated with clove and cinnamon essential oils showed several cells that were elongated and lysed. Similar results were reported by several other researchers previously [27,28].

As listed in Tables-1 and 2, clove buds and cinnamon bark harbor various essential oils that have different antibacterial activities. Clove bud contains 15-20% essential oil, dominated by eugenol (70-85%), eugenyl acetate (15%), and β-caryophyllene (5-12%) [7]. Eugenol is a bioactive compound with bactericidal damaging activities, such as holes in the envelope and bacterial cell deformities [29]. Cinnamon bark contains 0.5-1% essential oil consisting of cinnamaldehyde (63.69%), cinnamyl acetate (9.93%), and 1,8-cineole (8.75%) [8]. Cinnamaldehyde also has a bactericidal activity that can affect the permeability and integrity of the membrane and morphology of bacterial cells [27].

On the basis of our findings, clove and cinnamon essential oils have antibacterial activity and are equally effective against *K. pneumoniae* ATCC 700603 and ESBL-producing *E. coli* from chicken meat that are resistant to β-lactam antibiotics and *E. coli* ATCC 25922 that is not resistant to β-lactam antibiotics.

## Conclusion

This study reveals that clove and cinnamon essential oils have emerged as effective agents by showing high antibacterial activity against each bacteria tested, indicated by the inhibition zone diameter and MIC value. On the basis of the data, clove and cinnamon essential oils can be recommended as bioactive compounds to control the spread of ESBL-producing *E. coli* and *K. pneumoniae* isolates.

## Authors’ Contributions

EVG: Collected samples, conducted research in the laboratory, analyzed the data, and wrote the manuscript. DAW and ER: Delivered reagents and materials, analysis of the results, examined the data, and wrote and critically revised the manuscript. All authors read and approved the final manuscript.
